# Adaptor proteins mediate CXCR4 and PI4KA crosstalk in prostate cancer cells and the significance of PI4KA in bone tumor growth

**DOI:** 10.1038/s41598-023-47633-4

**Published:** 2023-11-23

**Authors:** Barani Govindarajan, Diego Sbrissa, Mark Pressprich, Seongho Kim, Arun K. Rishi, Ulka Vaishampayan, Michael L. Cher, Sreenivasa R. Chinni

**Affiliations:** 1 Department of Pathology, Wayne State University, School of Medicine, 9245 Scott Hall, 540 E. Canfield Avenue, Detroit, MI 48201 USA; 2 Department of Urology, Wayne State University, School of Medicine, Detroit, MI 48201 USA; 3 Department of Oncology, Wayne State University, School of Medicine, Detroit, MI 48201 USA; 4 Biostatistics and Bioinformatics Core, Wayne State University, School of Medicine, Detroit, MI 48201 USA; 5 Department of Oncology, University of Michigan, 7217 Rogel Cancer Center, Ann Arbor, MI USA

**Keywords:** Cancer, Urology

## Abstract

The chemokine receptor, CXCR4 signaling regulates cell growth, invasion, and metastasis to the bone-marrow niche in prostate cancer (PCa). Previously, we established that CXCR4 interacts with phosphatidylinositol 4-kinase IIIα (PI4KIIIα encoded by PI4KA) through its adaptor proteins and PI4KA overexpressed in the PCa metastasis. To further characterize how the CXCR4–PI4KIIIα axis promotes PCa metastasis, here we identify CXCR4 binds to PI4KIIIα adaptor proteins TTC7 and this interaction induce plasma membrane PI4P production in prostate cancer cells. Inhibiting PI4KIIIα or TTC7 reduces plasma membrane PI4P production, cellular invasion, and bone tumor growth. Using metastatic biopsy sequencing, we found PI4KA expression in tumors correlated with overall survival and contributes to immunosuppressive bone tumor microenvironment through preferentially enriching non-activated and immunosuppressive macrophage populations. Altogether we have characterized the chemokine signaling axis through CXCR4–PI4KIIIα interaction contributing to the growth of prostate cancer bone metastasis.

## Introduction

The CXCR4 is a chemokine receptor overexpressed in several types of cancers and serves as a key predictor of poor overall survival in cancer patients^1–3^. CXCR4 is transcriptionally regulated in PCa cells through the TMPRSS2-ERG fusion by androgens, along with other microenvironmental factors^4–6^. The activation of the CXCR4 receptor is shown to relay signals leading to proliferation, migration, invasion, and metastasis, promoting various stages of cancer progression^7–15^. This pathway is also responsible for the initial colonization of cancer cells to the stem cell niches in bone for the establishment of metastasis^13,16,17^. The CXCR4 and its ligand CXCL12 are known to regulate the trafficking of various immune suppressive cells to the bone tumor microenvironment, thus contributing to the disease progression and therapy resistance^18–20^. In this regard, we determined the novel downstream interactors of CXCR4 in PCa cells, using SILAC proteomic analysis, and found phosphatidylinositol kinase PI4KIIIα enriched in CXCR4 expressing PC cells in addition to expected G-protein coupled receptor signaling components^21^.

PI4K enzymes regulate the precursor steps of the Phosphatidylinositol (PI) metabolism by phosphorylating the D4 position of the myo-inositol headgroup of PI, generating PI(4)P. There is a total of four PI4K kinases: PI4KIIα, PI4KIIβ, PI4KIIIα, and PI4KIIIβ, each specifically localized to certain cellular organelles^22^. PI4KA (PI4KIIIα) is responsible for the production of PI4P at the plasma membrane. PI4P at PM is the source for maintaining stable levels of PI(4,5)P_2_, which is an essential PI for various cell functions and is depleted upon GPCR signaling involving Phoshpo Lipase C enzyme-mediated degradation^23,24^. PI4KA association with PM is evolutionarily conserved; studies from yeast to mammalian cells identified EFR3, a membrane-associated protein, and TTC7, a cytosolic protein, serves as an adaptor protein for PI4KIIIα targeting the PM^25–27^. Several other membrane proteins have also been recently shown to participate in dynamic PI4KIIIα PM targeting, these include FAM126A^28^, several chemokine receptors^21^, and KRAS^29,30^, and these membrane proteins directly bind with TTC7 and EFR3, and facilitate PI4KIIIα PM localization. The functional significance of these integral membrane proteins is to provide a scaffold for plasma membrane tethering of PI4KIIIα and induce its activity in the inner leaflet of the plasma membrane. Here we are extending our previous observations that chemokine receptor CXCR4 indirectly forms an endogenous complex with PI4KIIIα with adaptor proteins in prostate cancer cells, and this interaction is ligand-dependent thus, CLCL12/CXCR4 axis induces the production of PI4P. PI4KA is highly expressed in prostate cancer metastasis thus, knockdown in prostate cancer cells leads to significant inhibition of on bone tumor growth. Using a clinical resource, we demonstrate that PI4KA expression in human prostate cancer bone metastatic biopsies is associated with poor overall survival.

## Results

### CXCR4 directly binds PI4KIIIα adaptor proteins in PCa cells

**Figure 1:**
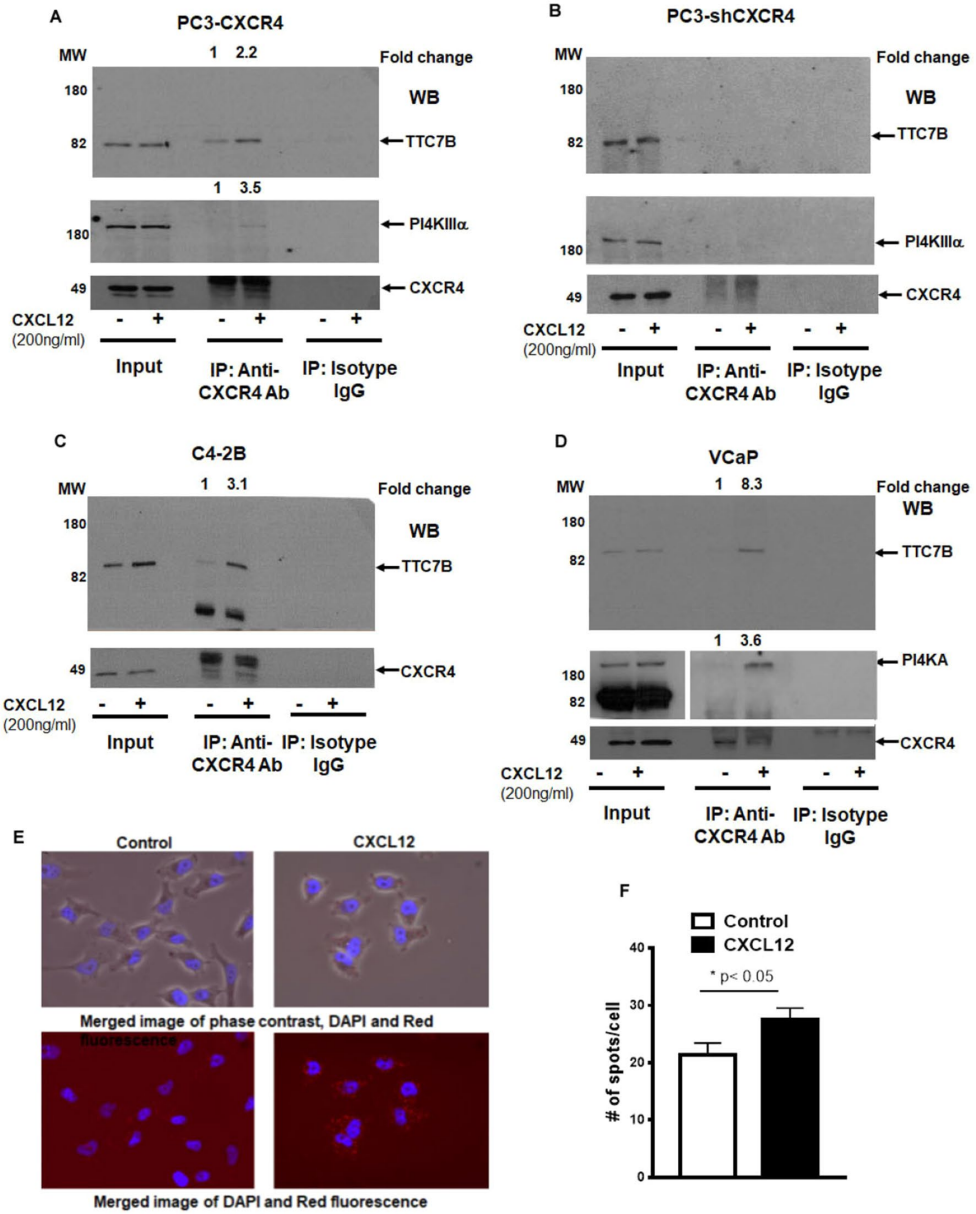
Chemokine receptor CXCR4 interacts with PI4KIIIα and its adaptor protein TTC7B in prostate cancer cells. (**A**,**B**) Co-immunoprecipitation showing pull-downs of CXCR4 and immunoblots of PI4KIIIα and adaptor protein TTC7B under basal and ligand-induced CXCL12 (200 ng/ml) conditions in (**A**) PC3-CXCR4, (**B**) PC3-shCXCR4, (**C**) C4-2B, and (**D**) VCaP prostate cancer cells. Grouped immunoblot images are either cropped from different areas of the same gel or from another gel run using the same protein lysate. (**E**) Co-localization of CXCR4 and TTC7B as measured by PLA assays in PC3-CXCR4 cells. (**F**) Spots refers to PLA signal showing the co-localization of CXCR4 with TTC7B. Graphs represent mean and standard error of triplicates performed over two experiments. The comparison was performed by Mann–Whitney test (*p < 0.05, in comparison to no ligand control).

We have previously shown the CXCR4 does not bind with PI4KIIIα but bind with PI4KIIIα adaptor proteins, EFR3B and TTC7B using transient transfection followed by immunoprecipitation^21^. EFR3B and TTC7B are key interacting proteins for PI4KIIIα plasma membrane localization and subsequent PI4P production. A triple transfection experiment shows the presence of CXCR4, PI4KIIIα, and its adaptor EFR3B exist as a complex in Cos-7 cells (Supplementary Fig. 1A). To determine the endogenous interaction of CXCR4 with PI4KIIIα adaptor protein TTC7B, we immunoprecipitated prostate cancer cells with CXCR4 and immunoblotted with TTC7B, PI4KIIIα and CXCR4. The data show that presence of complex between CXCR4, TTC7B and PI4KIIIα in PC3-CXCR4 and VCaP cells (Fig. 1A,C,D) demonstrating this interaction is predominant among prostate cancer cells. Treatment of prostate cancer cells with CXCL12 prior to immunoprecipitation shows 2.2 to 8.3 folds higher recruitment of TTC7B into CXCR4 complexes in PC3-CXCR4, C4-2B and VCaP cells and 3.5 to 3.6 folds higher recruitment of PI4KIIIα into CXCR4 complexes in PC3-CXCR4 and VCaP cells (Fig. 1A,C,D) suggesting CXCL12 activation of CXCR4 forms a complex with TTC7B and PI4KIIIα in prostate cancer cells. This complex is specific to CXCR4 as the IP experiment with CXCR4 knockdown PC3 cells show the absence of the complex (Fig. 1B). Previous studies show TTC7B interact with PI4KIIIα in cytosol, we also detected the presence of this complex in prostate cancer cells (Supplementary Fig. 1B). To quantitate and validate these findings, we performed a proximity ligation assay (PLA) by immunofluorescence imaging, using CXCR4 and TTC7B antibodies from different host species (Fig. 1E). There was an increase in localization between CXCR4 and TTC7B upon CXCL12 treatment, thus confirming a direct interaction of CXCR4 with TTC7B (Fig. 1F and Supplementary Fig. 1C).

### CXCL12 induces production of PI4P on the plasma membrane

**Figure 2:**
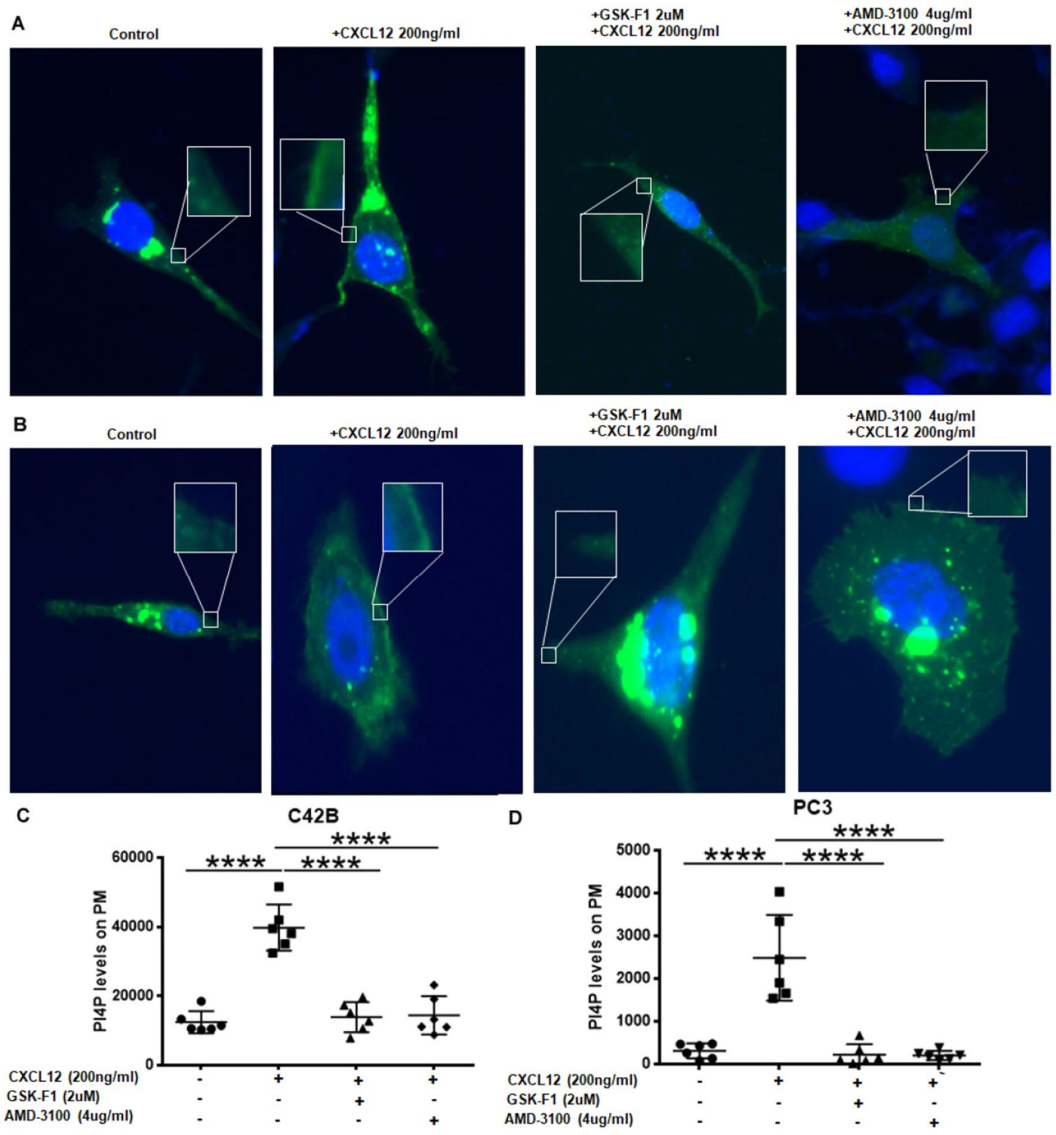
CXCL12 induction of CXCR4 increases PI4P production on the plasma membrane in prostate cancer cells. (**A**,**B**) Drug-dependent effects on PI4P production upon induction of CXCL12 as seen in immunofluorescence images after transfection with GFP-P4M-SidMx2 biosensors. Cells were serum starved overnight and drug treated for various conditions—control, GSK-F1 (2 μm), and AMD-3100 (4 μg/ml) and stimulated with CXCL12 (200 ng/ml) for 10 min in (**A**) C4-2B and (**B**) PC3 prostate cancer cells. (**C**,**D**) Changes in PI4P production is indicated as relative mean fluorescence value to control in box and whisker plots, after repeated plasma membrane fluorescence measurements of 5 to 6 different cells per condition. Comparisons were performed by one-way ANOVA followed by Tukey’s post-hoc comparisons in (**C**) C4-2B and (**D**) PC3 prostate cancer cells (****p < 0.0001).

To determine the impact of CXCL12/CXCR4 induced recruitment of TTC7B–PI4KIIIα complex, its activity was measured in prostate cancer cells. GFP-P4M-SidMx2 is a biosensor that binds to PI4P with high specificity^31^ was employed with prostate cancer cells to determine plasma membrane PI4P levels (Fig. 2A,B, Fig. S2). CXCL12 treatment increased the PI4P levels on the plasma membrane as a measure of increased GFP through SidMx2 binding to PI4P generated due to PI4KIIIα activity. Whereas, CXCR4 inhibitor AMD3100 treatment leads to inhibition of PM-associated GFP-P4M-SidMx2, suggesting that CXCL12/CXCR4 signaling generates PM PI4P production through recruitment of PI4KIIIα-TTC7B complex. In addition, the treatment of cells with GSK-F1, a specific inhibitor of PI4KIIIα also abrogated PM PI4P production as expected (Fig. 2C,D, Fig. S2). These data demonstrate that the CXCL12/CXCR4 signaling mediated activation of PI4KIIIα induces PI4P production on the plasma membrane.

### Loss of TTC7B inhibits both CXCR4 interaction with PI4KIIIα and chemokine mediated invasion

**Figure 3:**
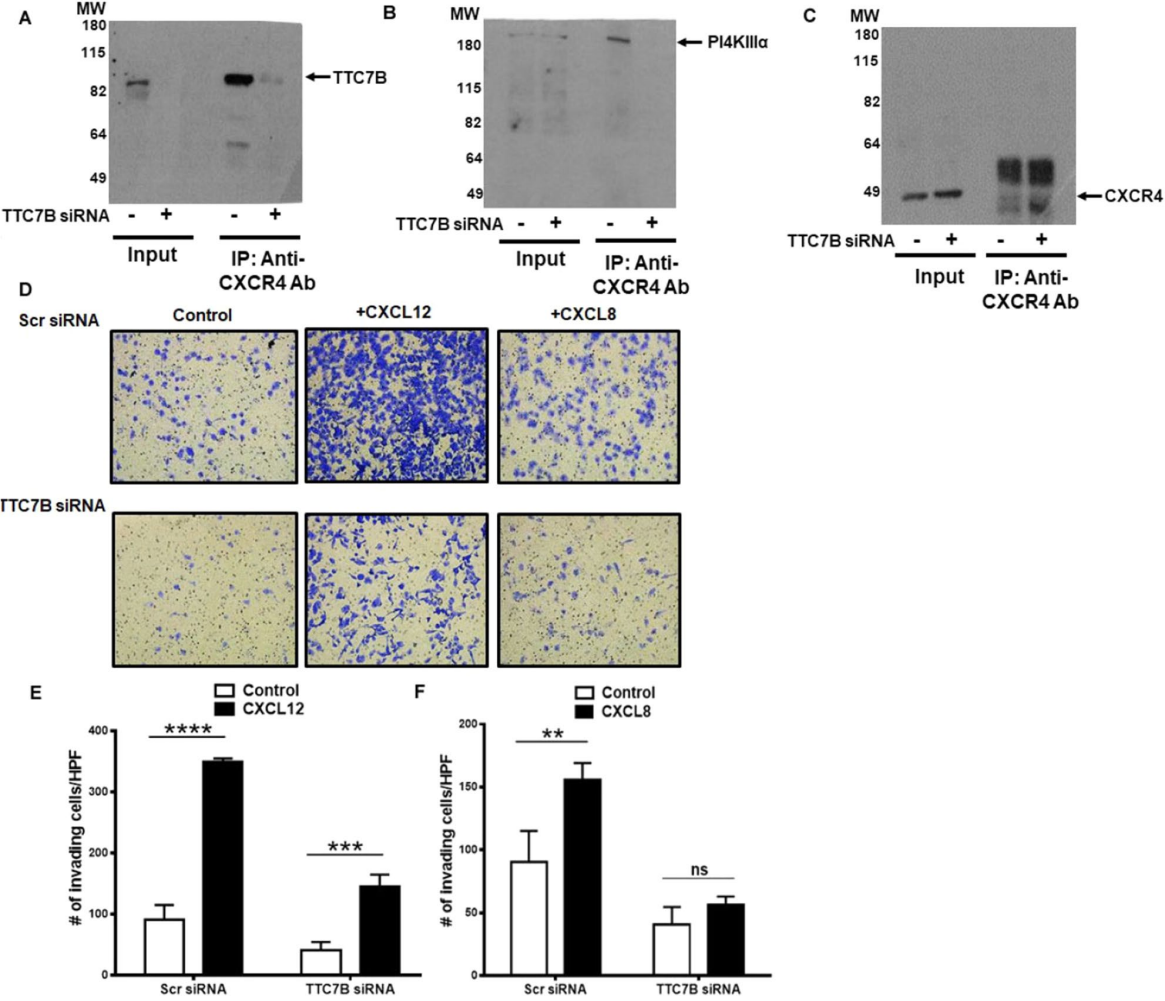
Adaptor protein TTC7B interacts with CXCR4 and also regulates invasion in PC3-CXCR4 prostate cancer cells. (**A**) TTC7B knock-down using siRNA (100 nM) in inputs and pull-down of CXCR4 in co-immunoprecipitation assays, showing immunoblots of (**A**) TTC7B, (**B**) PI4KIIIα and (**C**) CXCR4. (**D**) Matrigel invasion assays after treatment with TTC7B siRNA or Scramble siRNA (10 ×; upper and lower panels), under basal and ligand-induced CXCL12 (200 ng/ml), CXCL8 (50 ng/ml) conditions. Graphs represent mean and standard error of triplicates. Comparisons were performed using two-way ANOVA followed by Tukey’s post-hoc comparisons (ns, *p ≥ 0.05; **p < 0.01; ***p < 0.001; ****p < 0.0001, in comparison to no ligand control).

To determine the functional significance of the adaptor protein TTC7B in CXCR4 and PI4KIIIα interaction, we knock down TTC7B expression with siRNA. Immunoprecipitation studies show CXCR4 interaction with TTC7B and PI4KIIIα are inhibited upon TTC7B knockdown (Fig. 3A–C). We have previously shown that PI4KIIIα activity is localized to the invasive protrusions in prostate cancer cells as measured by PI4P immunostaining and regulates the multiple chemokines induced prostate cancer cell invasion as the receptors for chemokines are shown to interact with PI4KIIIα adaptor protein TTC7B^21^. As expected, both chemokines CXCL12 and CXCL8 induced chemoinvasion in scr siRNA transfected cells (Fig. 3D). In TTC7B siRNA transfected cells, both basal as well as chemokine-induced chemoinvasion is inhibited (Fig. 3E,F), suggesting that TTC7B regulates chemokine receptor-induced cancer cell invasion.

### PI4KA knockdown inhibits bone tumor growth

**Figure 4:**
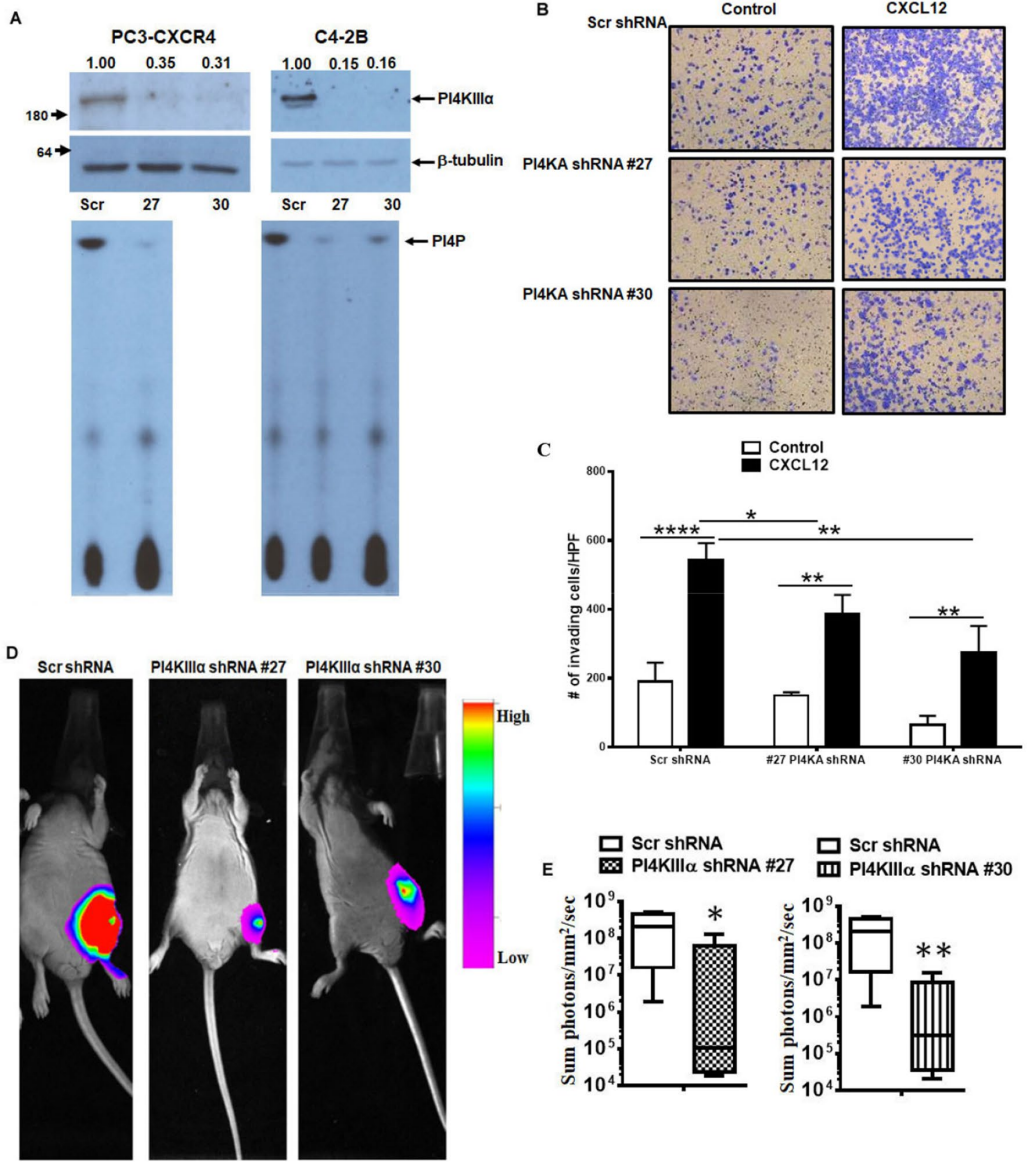
PI4KIIIα is involved in ligand-induced invasion in PCa cells and proliferation in-vivo. (**A**) immunoblots and lipid-kinase assay showing PI4KIIIα stable knock-down using lentiviral transduction in C4-2B and PC3-CXCR4 cells. (**B**) Matrigel invasion assays of stable PI4KIIIα knockdown PC3-CXCR4 cells under basal and ligand-induced CXCL12 (200 ng/ml) conditions. (**C**) Graphs represent mean and standard error of triplicates. Comparisons were performed using two-way ANOVA, followed by Tukey’s post-hoc comparisons (**p < 0.01; ****p < 0.0001, in comparison to no ligand control). Comparisons between CXCL12 treatment groups show p < 0.05 (*) between Scr shRNA vs #27 PI4KAshRNA and p < 0.01 (**) for Scr shRNA vs #30 PI4KA shRNA. (**D**) Intratibial injections in castrated nude mice of stable PI4KIIIα knockdown PC3-CXCR4-Luc cells, showing tumor burden using bioluminescence 39–41 days post-injection (Scr N = 6 and #27 and #30 N = 5). (**E**) Quantitation of in vivo luciferase data from Scr and PI4KIIIα knockdown groups. Comparisons were performed using Mann–Whitney test (*p < 0.05, **p < 0.01 in comparison to Scr control).

CXCL12/CXCR4 signaling promote initial establishment of prostate cancer colonization in bone microenvironment by actively competing with osteoblastic niche and these interactions along with cross talk with bone microenvironment factors promoting bone tumor growth^32^. Thus, to determine the role of PI4KA in CXCL12/CXCR4 signaling contributing to bone tumor growth, we have developed stable knockdown of PI4KA in PC3-CXCR4 and C4-2B cell with two independent PI4KA shRNA (#27 and #30) through lentiviral transduction. PI4KA expression and kinase activity is inhibited in both cell lines upon shRNA mediated stable knockdown (Fig. 4A). Matrigel invasion of both PI4KA shRNA knockdown cell lines is inhibited compared to Src shRNA cells in basal as well as CXCL12 induced cell invasion (Fig. 4B,C). To determine the significance of PI4KA knockdown on bone tumor growth, Scr and PI4KA knock down PC3-CXCR4 cells implanted in the mice tibiae. Luciferase imaging of bone tumors show that tumor growth is significantly inhibited (p < 0.05 for #27 and p < 0.01 for #30 PI4KA shRNA) in both PI4KA shRNA knock down cells over scr shRNA infected cells (Fig. 4D,E). Taken together, these findings show that the PI4KA signaling contributes to the growth of cancer cells in bone.

### PI4KA expression associates with PCa metastasis and tumor proliferation

**Figure 5:**
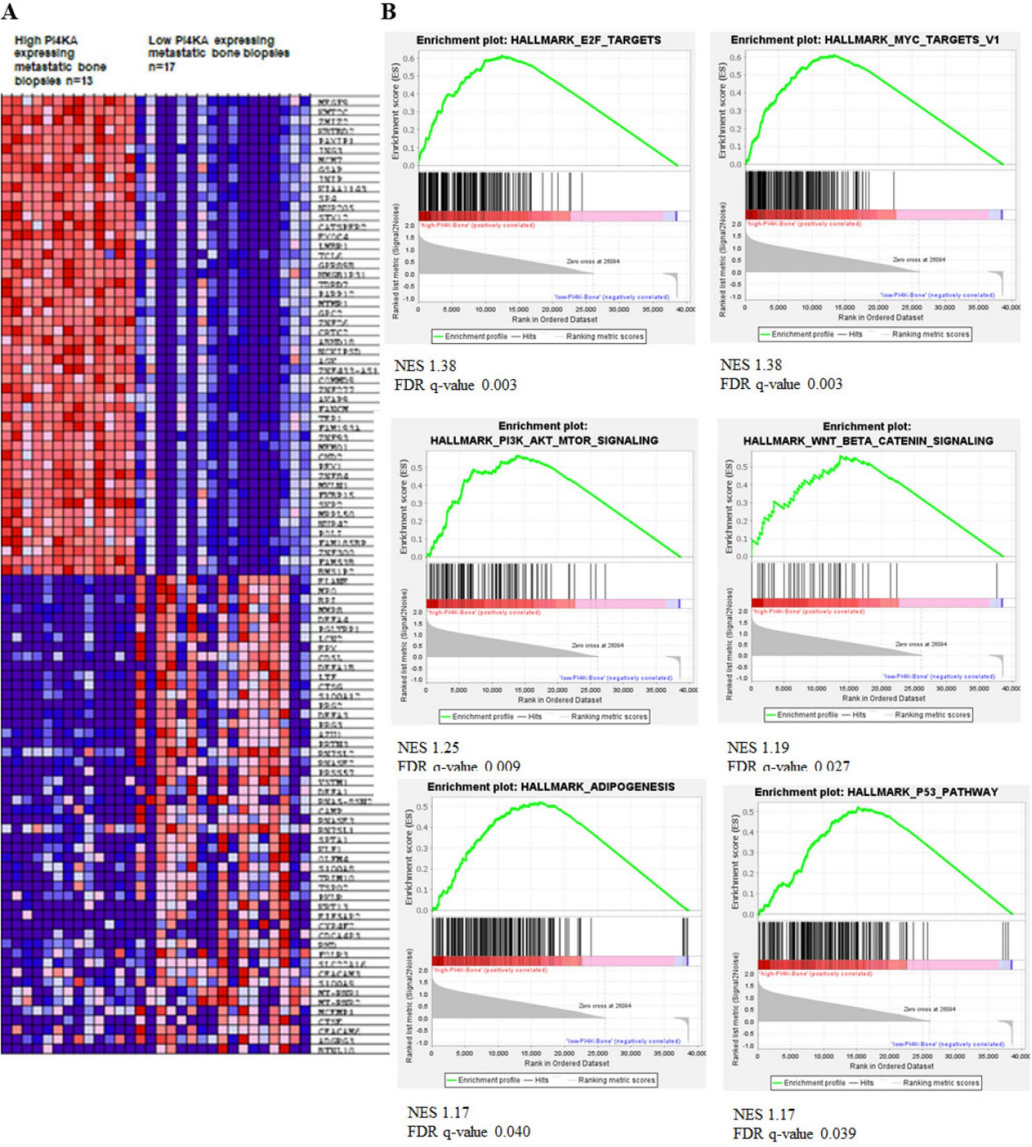
PI4KIIIα expression is associated with metastasis and proliferation in patient biopsies. (**A**) Heatmap representing individual expression levels of top and bottom 50 genes for each biopsy in expression dataset in metastatic bone biopsies of mHSPC patient samples. (**B**) Gene-sets enriched in bone biopsies expressing high PI4KA (n = 13), showing leading-edge pathways involved in cell-proliferation (FDR q-val < 0.05 and p < 0.05) (low PI4KA expressing bone biopsies (n = 17)). The samples were classified into low (mRNA expression < 22.5) or high (mRNA expression > 22.5) PI4KA.

Among the PI4P kinases, we found that PI4KA highly expressed in the metastatic prostate cancer compared to the matched primary tumors of PC patients^21^. We evaluated multiple NCBI datasets and found that PI4KA is significantly associated with metastatic disease, and in therapy resistance disease its expression is associated with neuroendocrine phenotype (Supplementary Fig. 3). To determine the impact of PI4KA expression on disease outcome in metastatic hormone sensitive PCa (mHSPC), we performed RNA-sequence analysis on 50 metastatic tumor biopsies, out of which 39 are bone biopsies, 10 lymph node and 1 liver biopsy. Clustering analysis of RNA-seq showed clear separation of gene expression profiles among mHSPC tumor biopsies (Supplementary Fig. 4). The bone met samples were categorized based on median high and low PI4KA expression and heatmaps of this analysis shows a set of genes commonly regulated across the tumor biopsy samples (Fig. 5A). The gene-set enrichment analysis (GSEA) of bone met biopsies showed enrichment of several leading-edge pathways involved in cell-proliferation in biopsies with high PI4KA expression (Fig. 5B and Supplementary Fig. 5). The top-most significant enrichment plots of some of the gene sets with FDR < 0.05 include the P53 pathway, Myc targets, PI3K-AKT-MTOR signaling, and Wnt-Beta-Catenin signaling. This data suggests that high PI4KA expression in bone mets is associated with cell proliferation, and this data is in line with our cell and animal bone xenograft experiment (Fig. 4B–D) that knock-down on PI4KA in PCa cells leads to inhibition of bone tumor growth.

### PI4KA expression correlates with poor overall survival (OS) along with an immunosuppressive phenotype in bone mHSPC

**Figure 6:**
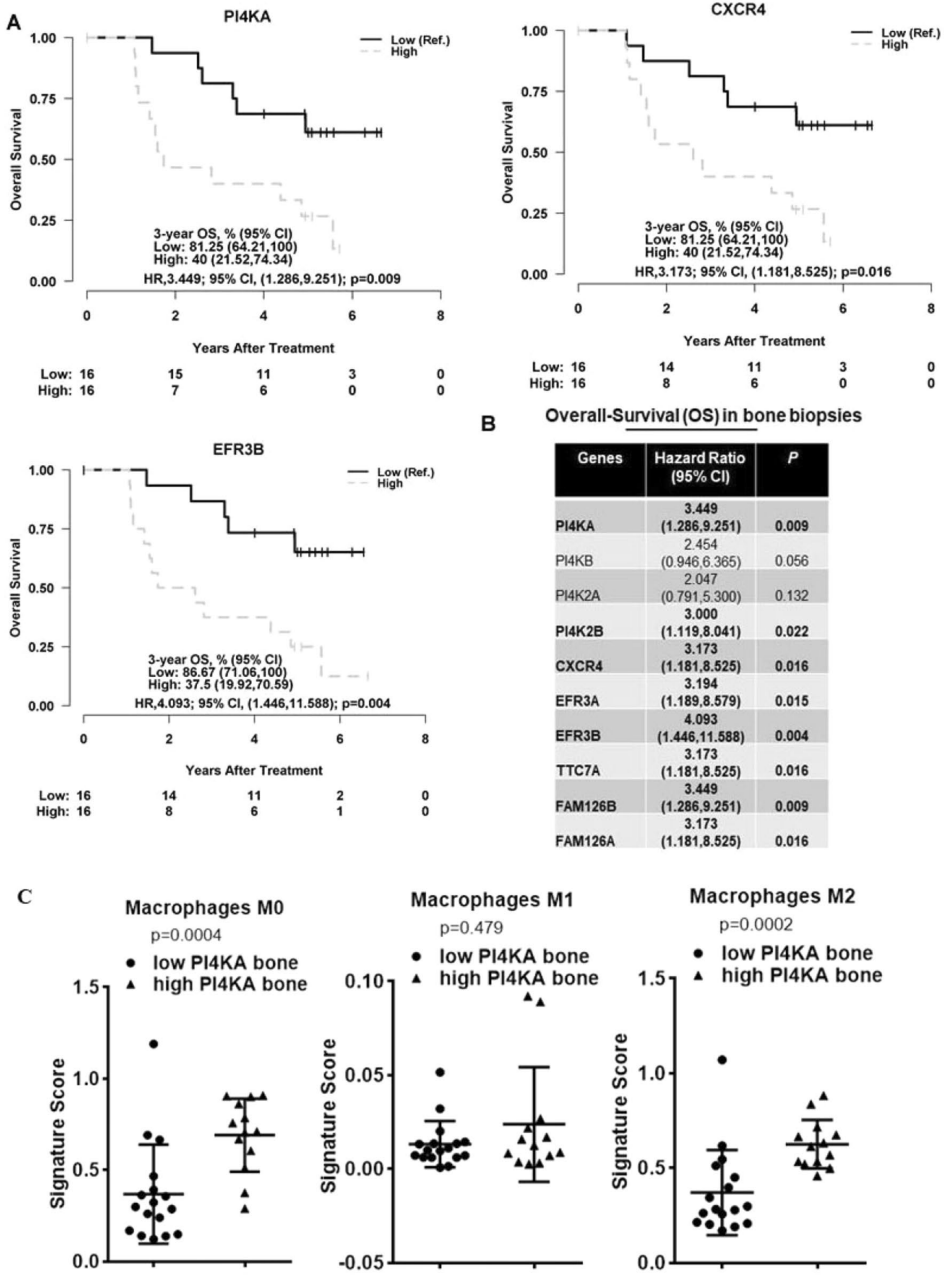
PI4KIIIα expression correlates with poor OS and an immunosuppressive phenotype. (**A**) Cox regression analysis of OS in metastatic bone biopsies of mHSCPC patients, shows significantly better OS in cohorts with low PI4KA and its associated proteins CXCR4 and EFR3B. Gene expression levels were dichotomized into high vs. low by their median. (**B**) Table of hazard ratios shows better OS in metastatic bone biopsies of mHSCPC patients with low PI4KA, its complexing proteins and CXCR4. Cox regression analysis was performed after gene expression levels were dichotomized into high vs. low by their median. (**C**) Cibersortx immune profile analysis of high PI4KA expressing bone biopsies shows high recruitment of non-activated macrophages (M0), and immunosuppressive macrophages (M2). Whereas there is no significant difference in immune-active macrophages (M1) between high or low PI4KIIIα expressing biopsies. Mast cells, natural killer (NK) cells, and CD4 memory cells are of higher expression in their resting state in high PI4KA expressing biopsies, whereas they are of higher expression in their active state in low PI4KA expressing biopsies. Comparisons were performed using Mann–Whitney test.

As prior data show that PI4KA expression is significantly higher in mHSPC samples, and its expression contributes to intraosseous growth in intratibial bone tumor model probably through the activation of tumor cell proliferative pathways in mHSPC. To determine the impact of PI4KA expression on clinical progression of the PCa, overall survival of the patients was evaluated based on tumor expression levels of PI4KA. The Kaplan–Meier graphs show poor OS in cohorts of bone biopsies, with high PI4KA and its upstream regulator CXCR4 expression (Fig. 6A), and this is also significant with the PI4KA adaptor proteins participating in the membrane localizations such as TTC7, EFR3 and FAM126 proteins (Fig. 6B). Among all the PI4P producing enzymes high tumor PI4KA and PI4K2B expression is associated with poor overall survival in mHSPC patients, whereas the expression of other two PI4P producing enzymes PI4KB and PI4K2A do not have statistical significance. This is not the case in soft-tissue biopsies, as no significant changes in OS was noticed https://www.msn.com/en-us/feedwith the expression of these genes (Supplementary Fig. 6).

We further characterized the immune landscape of bone tumor biopsies using differential gene expression analysis platform (CIBERSORTX)^33,34^ through their LM22 signature. Analysis of low and high PI4KA expressing metastatic bone biopsies show that high PI4KA expressing bone biopsies showed a more non-activated, immunosuppressive phenotype, with increased expression of M0 and M2 macrophages. Although the expression of M1 immune-active macrophages were similar between low and high PI4KA biopsies, the presence of M0 and M2 makes the environment more irresponsive to any anti-tumor immune activity (Fig. 6C). Interestingly other immune cells of significance in the profiling, such as mast cells, natural killer cells (NK), and T-cells CD4 memory cells, were of higher expression in their resting state in high PI4KA expressing biopsies (Supplementary Fig. 7). The selective presence of immunosuppressive cells in high PI4KA-expressing tumors implies that PI4KA activity contributes to tumor growth, and anti-PI4KA therapies have the potential to reverse suppressive phenotype in metastatic prostate cancers.

## Discussion

Here we characterized PI4KIIIα and its adaptor proteins as interactors of CXCR4 mediating CXCR4–CXCL12 induced cellular invasion and proliferation, conferring poor clinical outcomes in mHSPC patients. We hypothesized that PI4KIIIα binds to CXCR4 through its adaptor protein, and promote downstream signaling, thus playing a role in CXCR4–CXC12 mediated tumorigenicity through invasion and proliferation.

The initial proof of concept that, PI4KIIIα interacts with CXCR4 through its adaptor proteins EFR3B and TTC7B was supported by transfection followed by co-immunoprecipitation studies in cos-7 cells using transient expression in our previous study^21^. Here, we asked whether this interaction is present in prostate cancer cells and drives chemokine signaling. The data support this concept that in prostate cancer cells, endogenous interaction is present between CXCR4 and PI4KIIIα adaptor protein TTC7B. This interaction is observed with three different PCa cell lines with distinct genetic makeup with respect to AR and TMPRSS2-ERG fusion status. Thus, this endogenous interaction appears to be common among prostate cancer cells. Based on the immune precipitation data, we show that TTC7B binds to PI4KIIIα in the cytosol and upon CXCR4 activation this complex is recruited to the plasma membrane and binds to CXCR4. Presence of complex formation between CXCR4 and TTC7B in intact cells also determined with proximity ligation assay. Our data also show that EFR3B also binds with CXCR4 (Ref.^21^ and Supplementary Fig. 1), as EFR3 recruitment of PI4KIIIα is established earlier; together our data support the model that TTC7B and PI4KIIIα complex recruited to the CXCR4 and EFR3B complex on the plasma membrane, thus, CXCR4–EFR3B serves as a docking place for TTC7B–PI4KIIIα complex.

Next, we investigated functional significance of CXCR4–TTC7B interaction in promoting PI4KIIIα activity in PM. We used a novel PI4P probe by Hammond et al., that utilizes the P4M domain biosensor to detect the PI4P lipids, from *L. pneumophila* SidM^31^. This probe was characterized for its high specificity and efficiency in localizing to PI4P in live cells, compared to prior probes, and was shown to detect localization in organelles such as PM, Golgi and late endosomes. Another advantage of this probe for use in our study is that the probes are already validated^31^ to have just the appropriate amount of affinity to PI4P, in order to detect the fluctuating PI4P abundance on the PM. In our study the CXCL12 activated CXCR4 is widely studied to be on the PM, so we focus on the fluorescence fluctuations on the PM when studying this CXCR4–PI4KIIIα crosstalk. Chemokine CXCL12 induced a significant increase in the PI4P levels in PM presumably through TTC7B-P4KIIIα localization to PM. This increased localization to PI4P is abrogated in the presences of CXCR4 inhibitor and as expected specific PI4KIIIα inhibitors also abrogated SidM binding to PI4P suggesting chemokine signaling selectively activating PI4KIIIα activity on PM.

Our study contributes a new insight into the significance of CXCR4 induced activation of PI4KIIIα. CXCR4 overexpression enhanced the PI4P levels in the invasive projections in prostate cancer cells and abrogation of production through knockdown of PI4KIIIα leads to inhibition of chemokine induced PC cell invasion. Several members of chemokine receptors were shown to bind with PI4KIIIα adaptor proteins and translocate PI4KIIIα to PM to regulate its activity at PM. The common theme for chemokine receptor activation of PI4KIIIα is the binding to its adaptor protein for local production of PI4P for cellular invasion^21^. In support of this concept, we show that knockdown of TTC7B leads to inhibition of PI4KIIIα interaction with CXCR4 in plasma membrane and subsequent inhibition of its recruitment to PM. Similar to PI4KIIIα knockdown, TTC7B knockdown also significantly inhibited cellular invasion of prostate cancer cells suggesting that TTC7B adaptor mediated recruitment of PI4KIIIα to CXCR4 is critical for chemokine induced PC cell invasion. In addition, other chemokine CXCL8 induced cell invasion also inhibited in TTC7 knockdown cells suggesting TTC7B adaptor function regulating PI4KIIIα activity at membrane is common to other chemokine signaling. Previous studies including ours demonstrate that CXCR4 signaling contributes to prostate cancer cell colonization to bone tumor microenvironment and interact with osteoblastic niche for establishment of PC bone metastasis, herein, our data also demonstrate that PI4KIIIα knockdown significantly inhibited bone tumor growth suggesting CXCR4 downstream signaling through PI4KIIIα activation in PCa cells contributes to bone tumor growth. These observations are inline with critical cellular function of PI4KIIIα signaling in plasma membrane in maintaining PI(4,5)P_2_ synthesis, localizing certain PM associated proteins and maintaining PM cholesterol content^26^.

Here we provided the clinical significance of PI4KA expression in prostate cancer. Our previous study demonstrates that PI4KA expression is higher in metastasis compared to primary prostate cancer and in addition we showed that PI4KA expression is higher in metastasis with matched primary cancer tissue from patients^21^. Here in with analysis of two other data sets (GSE6919 and Ref.^35^) we show that the level of PI4KA expression correlates with PCa metastatic tumor and neuroendocrine differentiation phenotype. Altogether, the tumor expression data suggest PI4KA is associate with aggressive forms of PCa. These findings support our DEG analysis that identified metastatic bone biopsies expressing high PI4KA, to be enriched in cell proliferative pathways, potentially contributing to the metastatic tumor growth. The relevance of PI4KA is also evident from metastatic samples from hormone sensitive PCa patients, that exhibit poor OS and PSA progression in bone biopsies with higher expression levels. The COX regression analysis also showed significance in genes associated with PI4KA to be implicated in poor OS in bone biopsies. PCa also has a highly immuno-suppressive environment due to a multi-factorial regulatory, pro-tumorogenic and immunosuppressive environment, contributing to broad mechanisms of resistance^19,36–40^. Our results shed a new light on PI4KA expression in tumors and the status of immune cell profiles associated with tumors. Hihger PI4KA in tumors is associated with immunosuppressive phenotype of metastatic bone biopsies. Currently there are many ongoing trials that are testing immunotherapies with anti-CTLA-4, anti-PD-1/PD-L1, anti-CTLA-4 + anti-PD1/PD-L1, adenosine pathway inhibitors, bispecific antibodies and CART T cells, mostly in mCRPC and in some HSPC, mHSPC and CRPC cohorts^36^. PI4KA could be potentially used as a tumor-intrinsic biomarker for optimizing patient selection and responsive to specific treatment type.

## Materials and methods

### Cell culture

Prostate cancer cell lines PC3 and C42B (ATCC) were maintained in RPMI-1640 (Gibco-Invitrogen-Life Technologies), and supplemented with 10% heat-inactivated FBS (Hyclone, Fisher Scientific) and 1% P/S (50 units/ml penicillin, 50 μg/ml streptomycin, Gibco). VCaP (ATCC) cells were maintained in DMEM (ATCC), supplemented with 10% regular FBS (Cytiva, Hyclone, Fisher Scientific) and 1% P/S (50 units/ml penicillin, 50 μg/ml streptomycin, Gibco). C42B and PC3 stable, lentiviral generated cell lines were maintained in RPMI-1640 (Gibco-Invitrogen-Life Technologies) supplemented with 10% heat-inactivated FBS (Hyclone, Fisher Scientific), 1% P/S (50 units/ml penicillin, 50 μg/ml streptomycin, Gibco) and appropriate selection antibiotics (40 μg/ml blasticidin S for PC3-RFP and PC3-CXCR4 overexpressing cells; puromycin at 2 μg/ml for PC3 scrambled-shRNA or 24 μg/ml for PC3-CXCR4 shRNA knockdown cells; 40 μg/ml blasticidin S and 0.35 μg/ml puromycin for PC3-CXCR4 overexpressing and PI4KA or scrambled shRNA knockdown cells). Cell cultures were performed at 37 °C with 5% CO_2_. All cell lines were authenticated with STR analysis (Genomics core at Michigan State University, East Lansing, MI) and shown to have markers respective for each cell line as established by ATCC, and were tested for mycoplasma contamination with Venor-GeM mycoplasma detection kit (Sigma Biochemicals, St. Louis, MO).

### Lentiviral generation of stable cell-lines

Stably transduced PC3-CXCR4 and C4-2B cells with a knocked-down (GIPZ shRNA–PI4KA lentiviral construct) of PI4KA gene were produced using a Trans-Lentiviral Packaging Kit (Thermo-Fisher Scientific) according to manufacturer’s protocols. Briefly, GIPZshRNA–PI4KA lentiviral construct targeting the two independent sequences (#27 TAG ATC TCC AGT TGG CCA C (NM_058004: 4660-4678) and #30 TCA CTA ACT CCA CAT CGC T (NM_058004: 5516-5534)) of PI4KA mRNA (NCBI Reference Sequence: NM_058004.3) were obtained through GE Dharmacon (Lafayette, CO 80026)/Wayne State University Biobank Core Facility and used in a similar manner to transduce cells with infectious, replication incompetent lentiviral particles to generate stable PI4KA-knockdown cells using puromycin for selection of stable clones. Two clones were further characterized for PI4KA and knockdown and used in subsequent experiments.

### Western blot analysis

Total cellular proteins were extracted using RIPA buffer with 1 × Protease inhibitor cocktail (Roche, Indianapolis, IN). Protein was quantified using BCA protein assay (Pierce Biotechnology, Rockford, IL). Western blotting was performed using SDS-PAGE with gel transfer to a nitrocellulose membrane. Membranes were blocked in 5% BSA, probed with primary antibody in 5% BSA, and with secondary antibody linked with horseradish peroxidase, in 5% BSA. Enhanced chemiluminescence (ECL) substrate and autoradiography film was used to detect proteins. Densitometry was performed using image J software.

### Immunoprecipitation

Prostate cancer cells were grown in their respective complete media till 70% confluency. Transient transfections were performed if showing native interactions with over-expressions using 15 μg of plasmid and 15 μl Lipofectamine2000 Transfection Reagent (Life Technologies, Invitrogen) in 6 ml Opti-MEM media (Gibco). Cells were serum starved overnight, washed with PBS and treated with ligand CXCL12 (Peprotech, final [200 ng/ml]) for 10 min or left untreated. Cells were then lysed with 500 μl/100 mm-plate RIPA lysis buffer. Lysates were rotated in 4C for 15 min, and centrifuged at 15,000 rpm for 15 min. The supernatants were used to determine protein concentration using BCA protein assay kit. (ThermoFisher Scientific). 400–600 μg of protein lysate were rotated with 4 μg of antibody (CXCR4 AB1846 Millipore) at 4C overnight, followed by rotation with 40 μl of Pierce Protein A/G agarose (ThermoScientific) next day for 2 h at 4C. The samples were centrifuged at 5000 rpm for 30 s, beads were washed 3 times with RIPA wash-buffer and resuspended in denaturing sample buffer. The input samples along with the immunoprecipitation samples were heated at 100C for 5 min and immunoblotted as per Western blot analysis protocol.

### PI4KIIIα lipid kinase assay

In vitro PI4KIIIα lipid kinase assays were performed as described earlier^22^. Post kinase assay the chloroform-extracted PI(4)P product was separated by thin-layer chromatography (TLC) in n-propanol-2M acetic acid (65:35 v/v). PtdIns was visualized with I_2_ vapor following PI(4)P detection through autoradiography. PI4KIIIα activity was set as one-fold in control PC3 cells (PC3 scr) and compared with PI4KA (#27 and #30 PI4KA shRNA) manipulated cells.

### Cell proliferation and invasion assays

For cell invasion 24-well 8 μm transwells (Falcon) were coated with 37.5 μg Matrigel per insert, cells were seeded on the top of the chamber in serum-free media, along with chemo -attractants in the lower chamber in serum-free media. After 24 h cells were stained with 0.9% crystal violet and imaged for quantitation.

### Fluorescence microscopy

Cells were plated on coverslips coated with poly-l-lysine (Sigma) in a 6-well plate, and transfected with 2.5 μg of respective plasmids with lipofectamine. Cells were serum starved overnight; treated with either 2 μM GSK-F1 or 4 μg/ml AMD-3100 for 2 h and then ligand-induced with CXCL12 (200 mg/ml, Peprotech). After treatment, cells were fixed with 4% PFA with 0.2% glutaraldehyde at room temperature for 15 min. After aspiration, cells were further incubated with 50 mM NH4Cl in PBS and washed for 10 min, 3 times. Then cells were thoroughly washed with water, and mounted on slides using Vectashield with DAPI. Cells were imaged using a Leica DMi3000 B fluorescence microscope.

### Proximity ligation assay

Cells were plated on chamber slides and serum starved overnight. After ligand induction with CXCL12 (200 ng/ml), cells were fixed with 4% PFA and permeabilized with mild buffer 0.01% Tween-20 in PBS. The cells were further treated as per the Sigma Duolink DUO92101-1 kit. CXCR4-mouse and TTC7B-rabbit antibodies were used for primary incubation and observed for the presence of co-localization of CXCR4 and TTC7B using a fluorescence microscope.

### Intra-tibial bone tumor growth assessed by luciferase imaging

For in-vivo studies PC3-CXCR4 Scrambled shRNA Control and PC3-CXCR4 PI4KA shRNA (#27 and #30) cells were infected with Luciferase-2 lentiviral particles for in-vivo bioluminescence images of tumors. Castrated 4 to 5-week old male Nude mice (Taconic Farms, Germantown, NY) were used in this study. All the procedures including animal housing, surgery, imaging, the methods of anesthesia and euthanasia prior to tumor tissue analysis were performed as per the institutional animal care and use committee approved protocol. For intratibial implantation, 1.0 × 10^6^ cells in a 10 μl volume were injected per bone. Animals were imaged periodically to measure bone tumors. Luciferase imaging of tumors were performed with Carestream Invivo Xtreme system.

### Patient and clinical data

Pre-biopsy samples from patients with metastatic hormone-sensitive prostate cancer (mHSPC) were used for the clinical analysis in these studies. These patients participated in a multi-center trial conducted in 4 different centers in the US. An informed consent was obtained from all the patient prior to their participation in the trial. These men have no history of seizures, and have adequate marrow, renal and liver function, with a median age of 65. Clinical outcome data of the PSA response rate and Overall Survival statistics were obtained from this study for our analysis (Clinical trial: Identifier: NCT02058706). All methods were performed in accordance with the relevant guidelines and regulations.

Total RNA was extracted using the RNeasy midi kits (Qiagen) along with ON-Column DNase digestion (Qiagen), as described below, and sequenced at University of Michigan Genomics core. A total of 39 bone biopsies and 23 soft-tissue biopsies were used in this study after RNA quality was confirmed, and RNA-sequencing (RNA-seq) was performed. The GSEA analysis was performed on the resulting RNA-seq TPM data. Cibersortx machine-learning tool was also utilized to identify immune expression profile using the LM22 signature.

### Gene set enrichment analysis

Pathway enrichment was performed using GSEA software (version 4.1). The Hallmark pathway dataset was downloaded from the MsigDB database of the GSEA website. The median high vs low-PI4KA expression profile of biopsies from mHSPC patients, and the attribute files were enriched and analyzed by default weighted enrichment statistics. The number of permutations was set to 1000.

### Gene expression omnibus database

GSE6919 from the public database of GEO-NCBI was used to analyze expression profiles from tissue biopsies that represent prostate cancer progression from normal tissue free of any alteration, normal tissue adjacent to primary tumor, primary tumor to metastatic prostate tumor samples. The total 504 samples from these different states were extracted, by downloading the platform and matrix files from the GSE6919 database. Data in the files were analyzed using GraphPad Prism 6.

### Statistical analysis

GraphPad Prism 6 and R were used to assess statistical significance. Distributions of continuous outcomes were checked for normality and, if needed, non-parametric approaches were used. Comparisons between two groups were performed using unpaired *t*-test or Mann–Whitney test. For three or more groups, one- or two-way ANOVA was used, followed by Tukey’s post-hoc pairwise comparisons. Survival outcomes were graphically summarized using Kaplan–Meier curves, and a log-rank test and Cox regression analysis were used to compare between groups. If a p value was less than 0.5, it was considered statistically significant. *p < 0.05. **p < 0.01. ***p < 0.001. NS stands for “not significant”.

### Ethics approval and consent to participate

The animal studies were performed in facilities monitored by division of laboratory animal resources (DLAR) at Wayne State University. The DLAR facility’s PHS Animal Welfare Assurance number is A3310-01, and the assurance is valid until February 28, 2026, USDA registration number is 34-R-0014. The University’s AAALAC file number is 393 and received full accreditation notification on March 9, 2021, and valid for 3 years. All the mice experiments were performed in accordance with institutional guidelines and ARRIVE guidelines 2.0.

Prostate cancer patient biopsies are collected under the approval of protocol 2013-083 from Institutional Review Board (IRB) at Wayne State University. Wayne State University Human Research Protection Program (HRPP) is fully accredited by the Association for the Accreditation of Human Research Protection Programs Inc. (AAHRPP). The institutes Federal Wide Assurance Number is FWA00002460. Informed consent was obtained from all the patients and research work was performed in accordance with approved IRB protocol. All methods were performed in accordance with the relevant guidelines and regulations.

### Supplementary Information

Supplementary Figures.

## Data Availability

The data sets generated during this study are in the process of depositing into the NCBI/GEO resource. Once this process is completed the unique geo identifier will be provided to the journal. The other data sets are available from the corresponding author on reasonable request.
