# Supplementary material for: Adaptor proteins mediate CXCR4 and PI4KA crosstalk in prostate cancer cells and the significance of PI4KA in bone tumor growth
Source: Sci Rep. 2023 Nov 23;13:20634. doi: 10.1038/s41598-023-47633-4 (PMC10667255; doi:10.1038/s41598-023-47633-4)
Supplement: Supplementary file 1 — Supplementary Figures. [file 41598_2023_47633_MOESM1_ESM.docx]

**Supplementary Information:**

**Figure S1.** A) CXCR4 interacts with PI4KIIIα in the presence of adaptor protein EFR3B. A) immunoblot showing triple transfections of tagged-proteins HA- PI4KIIIα, EFR3B-C-EGFP and Myc-CXCR4 in Cos-7 cells. Co-immunoprecipitation showing pull-down of Myc-CXCR4 and immunoblot of HA-PI4KIIIα; along with pull-down of HA-PI4KIIIα and immunoblot of Myc-CXCR4. B) TTC7B and PI4KIIIα complex in C4-2B prostate cancer cells. C). Control experiments for PLA: no primary antibody control and either CXCR4 or TTC7B antibody alone included in the experiment.


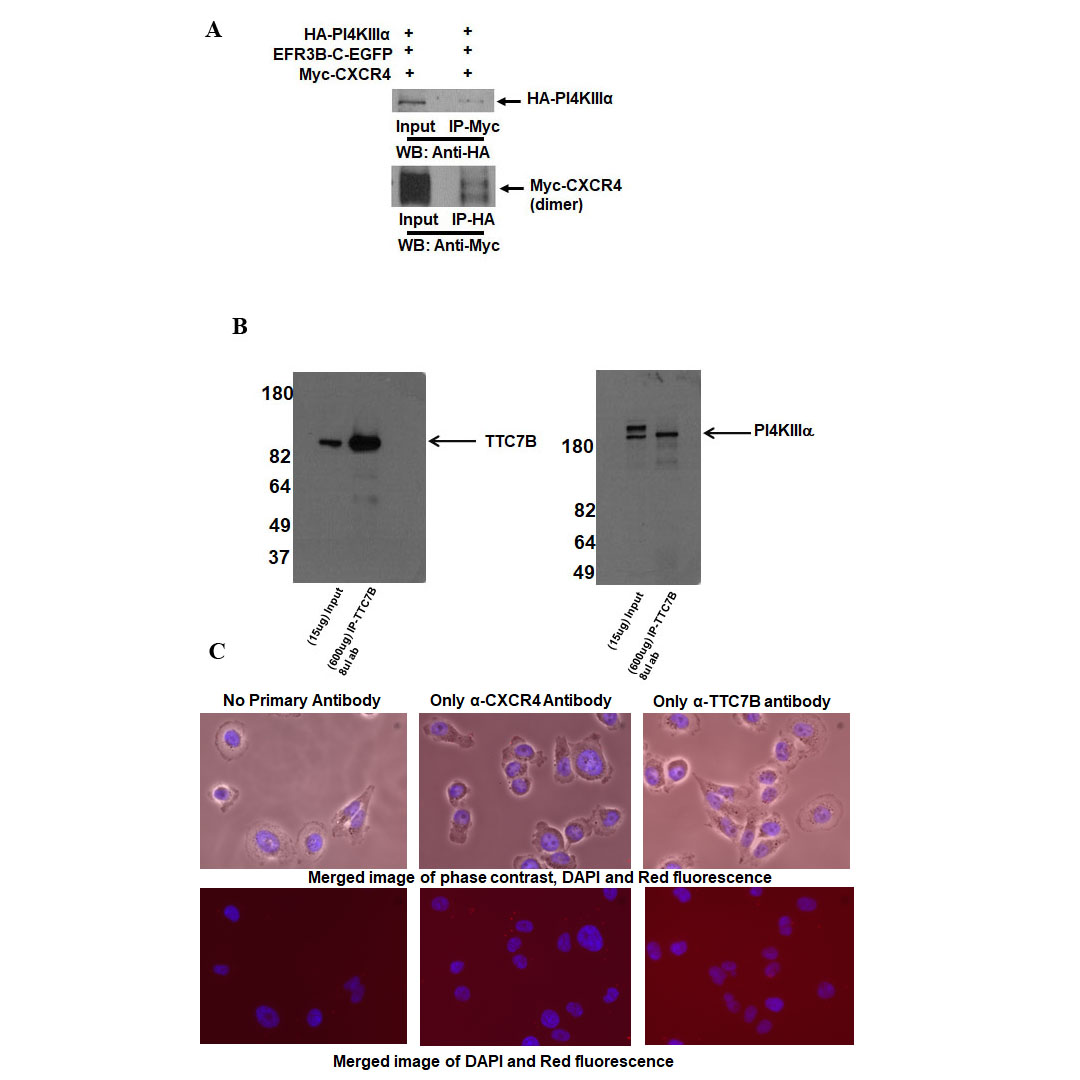


**Figure S2**. CXCL12 induction of CXCR4 increases PI4P production on the plasma membrane in VCaP prostate cancer cells. Drug-dependent effects on PI4P production upon induction of CXCL12 as seen in immunofluorescence images after transfection with GFP-P4M-SidMx2 (2215ng/ul) biosensors. Cells were serum starved overnight and drug-treated for various conditions- control, GSK-F1 (2um), and AMD-3100 (4ug/ml), and stimulated with CXCL12 (200ng/ml) for 10 minutes. Changes in PI4P production is indicated as relative mean fluorescence value to control in box and whisker plots, after repeated plasma membrane fluorescence measurements of 5 to 6 different cells per condition. Comparisons were performed using One-way ANOVA followed by Tukey’s post-hoc comparisons (****, *p*<0.0001).


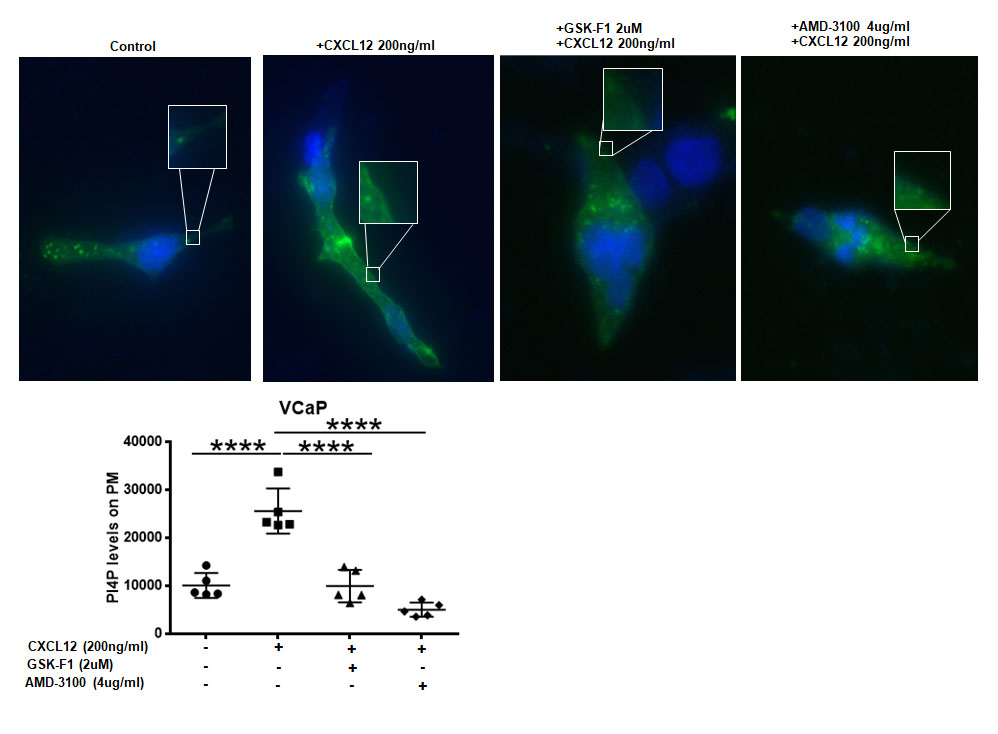


**Figure S3**. Top-panel) Expression analyses show PI4KA and CXCR4 are highly expressed in metastatic samples from expression arrays of normal and prostate tumor tissues (from NCBI GEO series GSE6919). Comparisons were performed by Mann-Whitney tests. Bottom-panel) Expression analysis show PI4KA and EFR3B are highly expressed in CRPC- neuroendocrine from whole exome sequencing of CRPC-adenocarcinoma and CRPC-neuroendocrine prostate tumor biopsies (from Beltran et al^41^). Comparisons were performed by Mann-Whitney test.


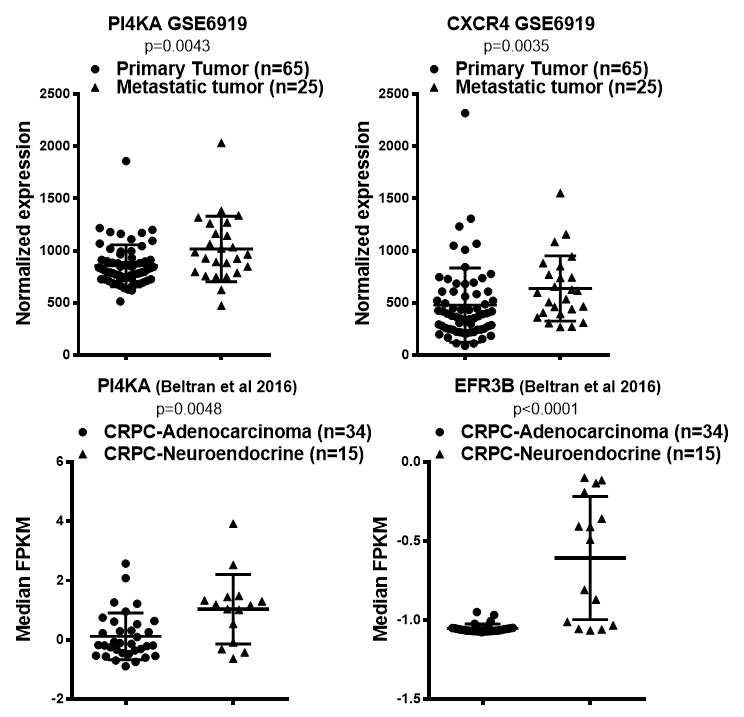


**Figure S4**.Clustering analysis of metastatic biopsy specimens from bone (n=32), liver (n=1) and lymph node (n=17). The supervised clustering was performed by partial least-squares discriminant analysis (PLS-DA) using gene expression data generated from RNA-seq analysis.


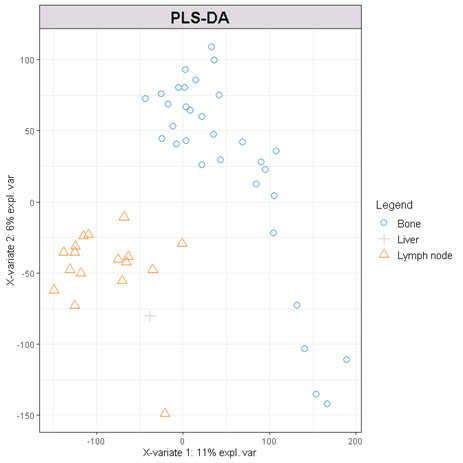


**Figure S5**. GSEA enrichment showing leading-edge pathways enriched in high PI4KA compared to low PI4KA metastatic bone biopsies from mHSPC patients. These top-most enriched pathways are associated with cell-proliferative characteristics in the high PI4KA expressing cohort.


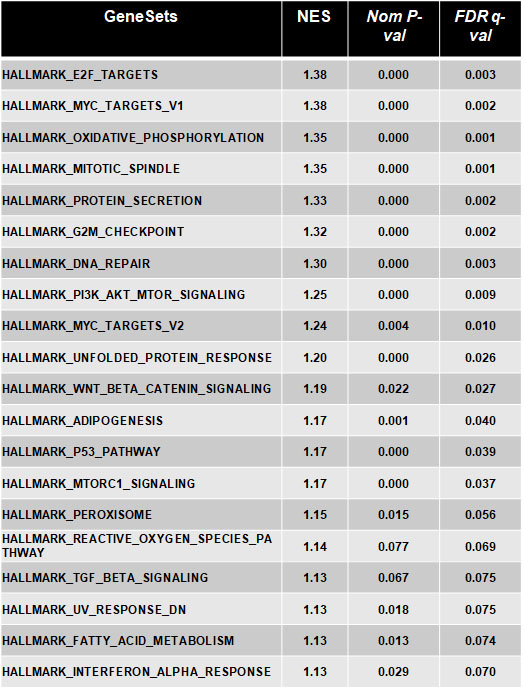


**Figure S6**. Table of hazard ratios showing no significant OS in metastatic soft-tissue biopsies of mHSCPC patients with low PI4KIIIα, its complexing proteins and CXCR4. Cox regression analysis was performed after gene expression levels were dichotomized into high vs. low by their median.


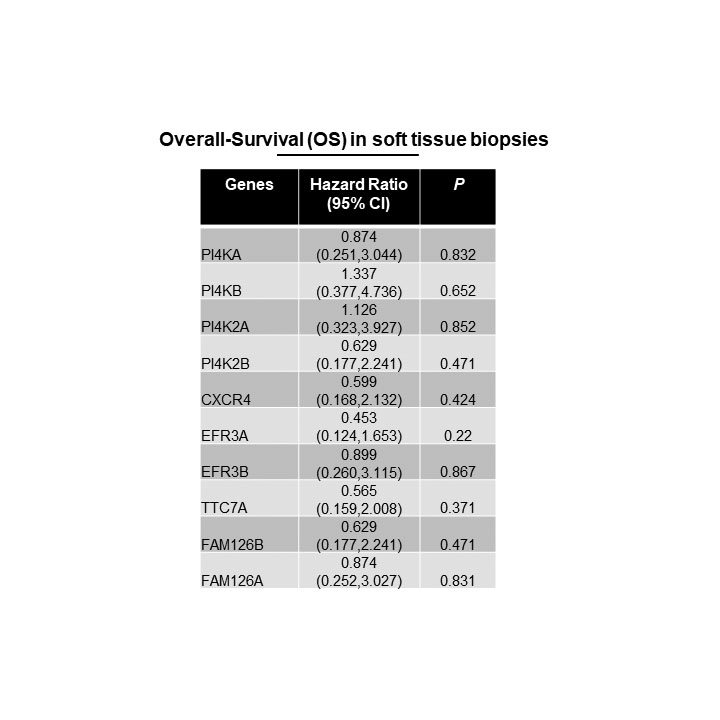


**Figure S7**. Cibersortx immune profile analysis of high and low PI4KA bone tumor biopsies showing several types of immune cells. Comparisons were performed by Mann-Whitney test.

**
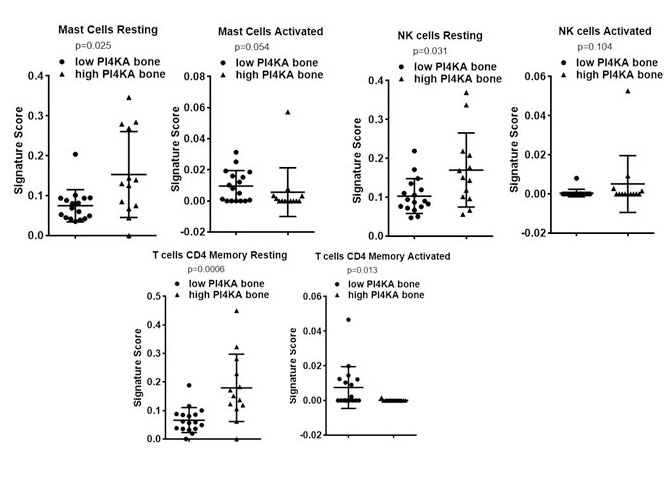
**

Raw data of Western blot analysis of chemiluminescence detection on photographic film. In all images the highlighted box portion of the data presented in respective figures in the manuscript. Whole photographic film or portion of film covering the entire or part of the blot were shown in the raw data figures. In some experiments membranes were cut and probed with antibodies and respective chemiluminescent detection of cut membranes were shown.

Figure 1ABCD gel images:

Figure 3ABC gel images:


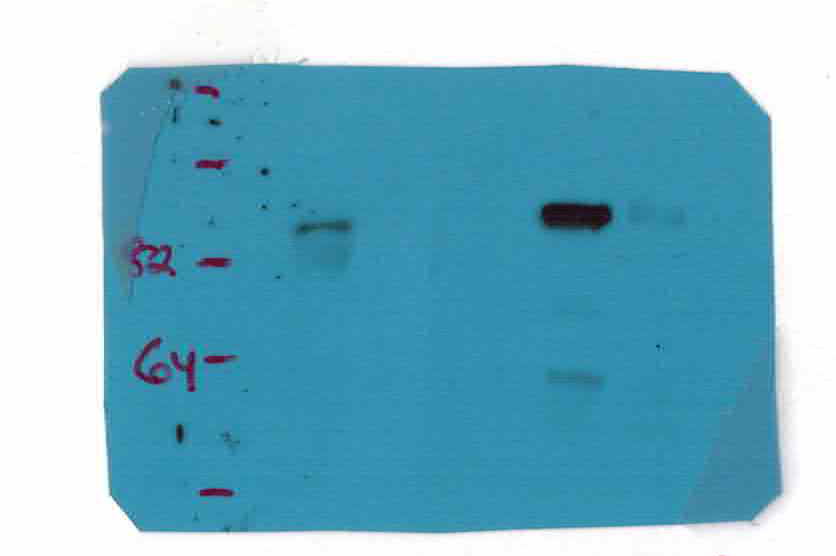

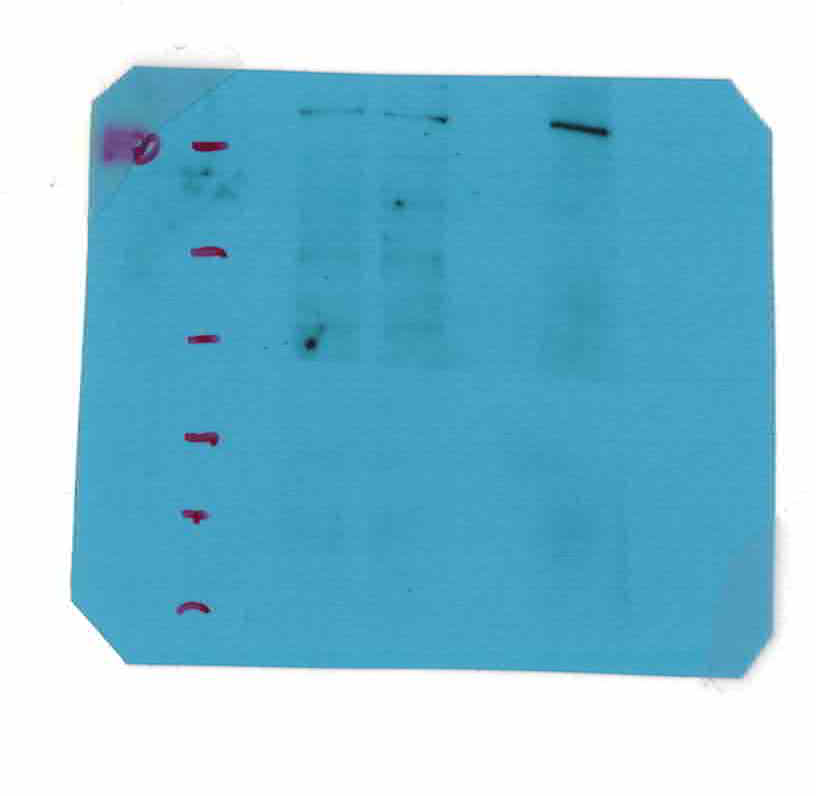

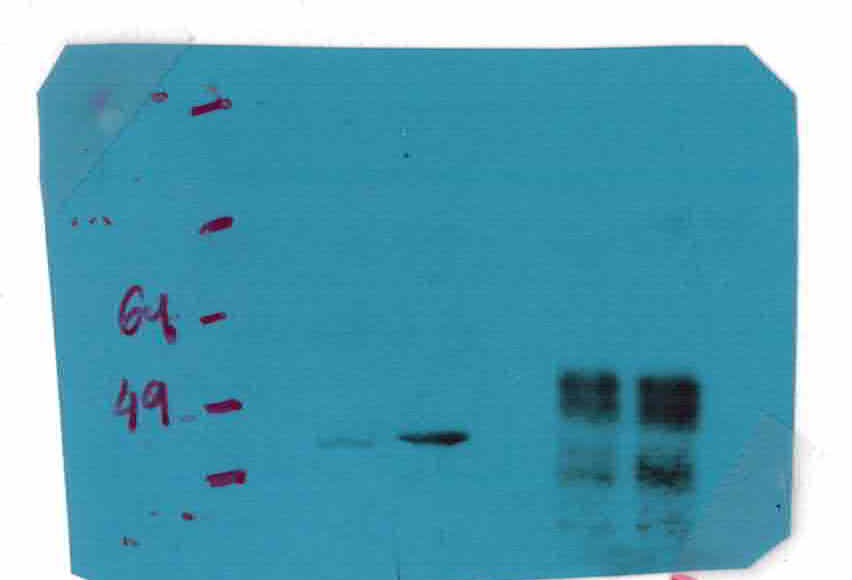


PI4KIIIα

CXCR4

TTC7B

Figure 4 gel images:

Supplementary Figure 1 A gel images:


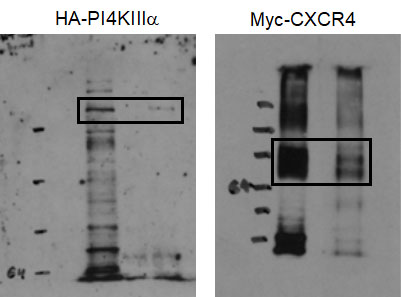


Supplementary Figure 1 B gel images:
